# Preserved Menstruation After Chemoradiotherapy in Stage IIIC1 Cervical Cancer: A Unique Case

**DOI:** 10.3390/jcm15041550

**Published:** 2026-02-15

**Authors:** Georgia Ilia, Athanasios Thomopoulos, Dimitrios Chronas

**Affiliations:** 1 Department of Obstetrics and Gynecology, University Hospital of Basel, 4031 Basel, Switzerland; 2 Department of Obstetrics and Gynecology, Spital Zollikerberg, 8125 Zurich, Switzerland; dimitrios.chronas@spitalzollikerberg.ch; 3 Departemet of Clinical Sciences, Medical School, University of Nicosia, Nicosia 2408, Cyprus; athanasios.thomopoulos@hirslanden.ch

**Keywords:** oncofertility, cervical cancer FIGO IIIC1, cervical cancer treatment, ovarian transposition, preserved menstruation

## Abstract

**Background**: In young women with cervical cancer, fertility preservation remains challenging, as chemoradiotherapy can severely compromise ovarian reserve and endometrial function. Although ovarian transposition prior to pelvic radiotherapy is well established in early-stage disease, evidence regarding ovarian and endometrial outcomes in advanced stages, particularly in the International Federation of Gynecology and Obstetrics (FIGO) stage IIIC1, remains extremely limited. **Case Presentation**: We report the case of a 31-year-old nulliparous woman with a histopathologically confirmed FIGO IIIC1 cervical squamous cell carcinoma who underwent a lateral ovarian transposition followed by external beam radiotherapy (ERBT) of the pelvis and interstitial high-dose-rate (HDR) brachytherapy combined with five cycles of cisplatin-based chemotherapy. A detailed dosimetrical analysis demonstrated extremely low ovarian radiation exposure (mean dose < 2 Gy bilaterally). Menstruation resumed seven months after treatment completion, with regular 27–30-day cycles. A day-3 hormonal assessment showed a partial preservation of the ovarian reserve, and the pelvic ultrasound confirmed a thickness of 7 mm in the proliferative phase, implying endometrial function despite full-dose pelvic irradiation. **Conclusions:** To our knowledge, this is a very unique case of preserved menstruation after ovarian transposition and chemoradiotherapy for FIGO IIIC1 cervical carcinoma. This case challenges the conventional assumptions regarding ovarian failure and endometrial destruction in such cases, suggesting that reproductive potential may occasionally be retained. Although fertility remains a challenging point, this case report underscores the need for individualized counseling and prospective oncofertility research.

## 1. Introduction

Cervical Cancer is the fourth most common cancer among women worldwide, with approximately 0.6 million new cases and 0.3 million deaths annually, accounting for 6.9% of the total cancer burden [1,2]. It has a prevalence of 13 in 100,000 women globally, with significant variation between high-income and low-income countries, as reported in a 2018 world population study [3]. The age distribution of cervical cancer rises after 25 years, with a distinct peak in women between 30 and 39 years of age [1,3]. The vast majority of cervical cancer cases are caused by the oncogenic activity of human papillomavirus (HPV), particularly types 16, 18, 31, 33, 45, 52, and 58, which account for more than the 90% of the invasive squamous cervical carcinoma cases worldwide [4]. Although cytological screening programs and the widespread use of HPV vaccination are established, a significant proportion of women diagnosed with cervical malignancy are nulliparous women under 45 years old who wish to preserve their fertility [5]. The treatment of cervical cancer depends on its stage. Advanced metastatic stages with positive lymph nodes typically require radical hysterectomy with lymphadenectomy or a combination of radiotherapy and chemotherapy [6,7]. In young women with advanced stages, fertility preservation remains a challenge, as both radiotherapy and chemotherapy can severely compromise ovarian reserve and endometrial function [8]. Fertility preservation can be achieved through ovarian transposition (OT). Fertility-preserving approaches such as ovarian transposition before pelvic radiotherapy are well described in early-stage cervical cancer, with meta-analyses reporting ovarian function preservation in approximately 60% of women exposed to EBRT (external beam pelvic radiotherapy) ± brachytherapy [9,10]. Data on endometrial function after chemoradiotherapy in advanced stages, particularly FIGO IIIC1, remain extremely limited. The available literature primarily focuses on ovarian function preservation, with very few reports addressing post-treatment menstruation. We report a unique case of preserved menstruation following ovarian transposition and definitive chemoradiotherapy for FIGO IIIC1 cervical cancer, integrating surgical details, ovarian radiation dosimetry, endocrine evaluation, and uterine imaging findings. This case provides insights into the potential for residual ovarian and endometrial function after chemoradiotherapy in FIGO IIIC1 disease. To the best of our knowledge, this is the first published case of successfully preserved regular menstrual cyclicity after OT followed by concurrent chemoradiotherapy (CCRT) for FIGO IIIC cervical carcinoma.

## 2. Case Presentation

**Figure 1:**
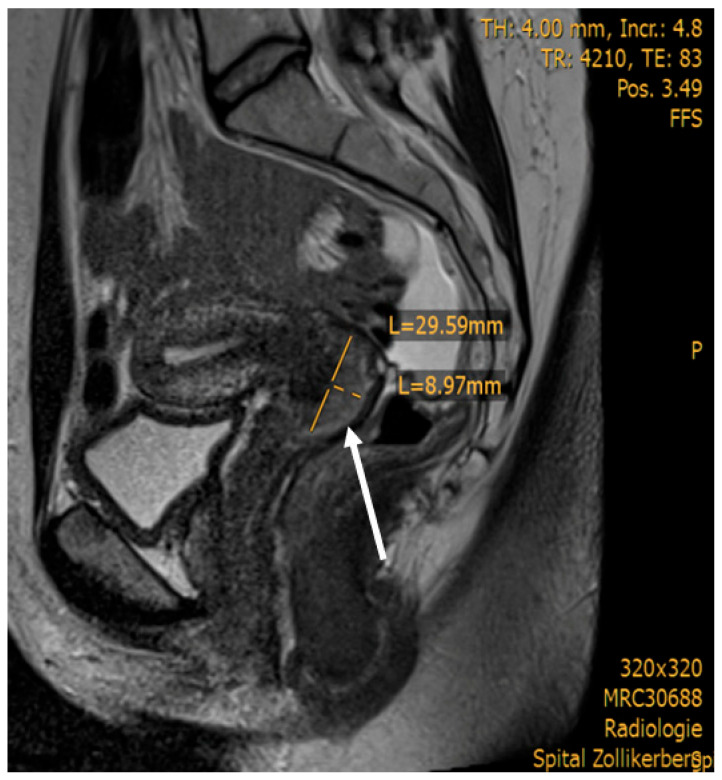
Sagittal MRI demonstrating tumor extent. Cervical carcinoma measuring 30 × 9 mm, located along the dorsal circumference of the cervix uteri, extending from the 9 o’clock to 4 o’clock positions (white arrow).

**Figure 2:**
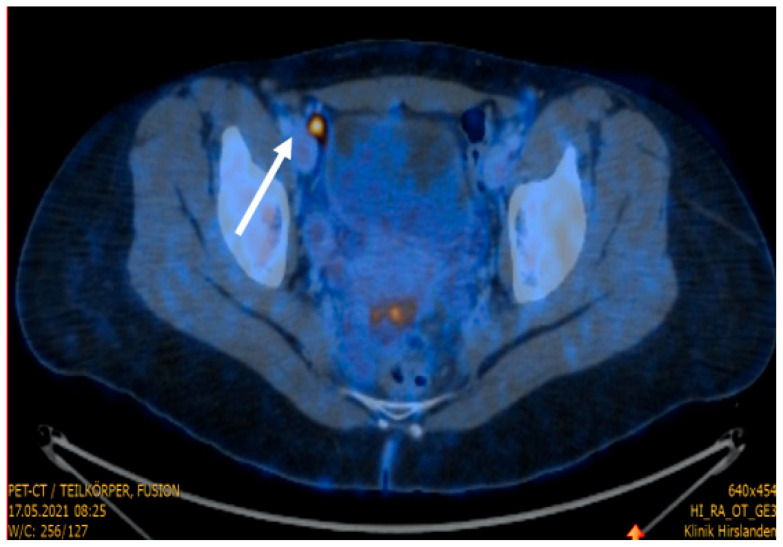
PET/CT showing a metabolically active lymph node. A centrally hypodense, metabolically active lymph node in the right external iliac region (SUVmax 9.5), measuring 12 mm in transverse diameter (white arrow).

We report the case of a 31-year-old nulliparous woman referred to our hospital for treatment of histologically confirmed squamous cell carcinoma of the cervix, positive for high-risk human papillomavirus (HPV) subtypes 58 and 59. There was no record of HPV vaccination in her medical history. The patient initially presented with a three-month history of postcoital bleeding. Her family history was unremarkable for cancer disease. Preoperative imaging, including pelvic Magnetic Resonance Imaging (MRI) and Positron Emission Tomography–Computed Tomography (PET-CT), revealed a ca 3 cm carcinoma of the cervix uteri and a suspicious metastatic lesion in the right external iliac lymph node (Figure 1 and Figure 2).

The patient underwent laparoscopic staging with resection of the suspicious enlarged right iliac lymph node (frozen-section examination) and a bilateral salpingectomy. A resection of the para-aortic lymph nodes below the origin of the inferior mesenteric artery, despite the negative MRI and PET CT, was also performed to define the radiation field extension. Laparoscopic staging was performed, as imaging-based staging can understate para-aortic disease and have false negative rates for microscopic para-aortic metastases [11].

**Figure 3:**
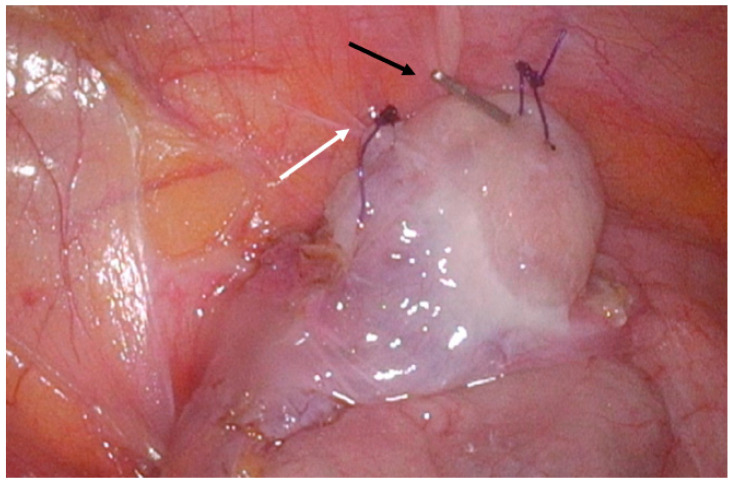
Laparoscopic ovarian transposition and fixation. The ovary is secured to the paracolic gutter at the L2–L3 level using three intracorporeally tied Vicryl sutures (white arrow). Radiopaque clips were applied to each ovary to facilitate postoperative localization (black arrow).

To preserve ovarian function, the patient underwent a bilateral OT. Both ovaries were mobilized and repositioned to their respective paracolic gutters, outside the radiation field. The infundibulopelvic ligaments were anchored to the corresponding lateral abdominal walls with three single interrupted 2-0 Vicryl sutures per side to prevent bowel herniation. Radiopaque clips (two on the right side and one on the left) were applied to each ovary (Figure 3). The ovarian transposition aimed to maintain hormonal function and offer the potential for future fertility preservation. Histopathological analysis confirmed grade 3 squamous cell carcinoma (FIGO IIIC1).

Postoperatively, she received EBRT over a period of six weeks, with 45 Gy in 25 × 1.8 Gy, which was augmented in the extended primary tumor region with an additional 9 Gy boost in 5 × 1.8 Gy, resulting in 54 Gy of radiation in the uterus, combined with five cycles of cisplatin-based chemotherapy. The dose-volume histogram (DVH) reported a planning target volume (PTV) pelvis mean: 49.4 Gy and PTV boost pelvis mean: 54.1 Gy. The entire uterus was encompassed within the PTV. Consequently, all uterine substructures received the full prescribed dose without any sparing. The ovarian doses were very low (right ovary mean dose: 1.2 Gy/max dose: 1.9 Gy; left ovary mean dose: 1.1 Gy/max dose: 1.7 Gy). The distance from the lower pole of the right ovary to the pelvic PTV was 4.0 cm, while the distance from the lower pole of the left ovary to the pelvic PTV was 4.6 cm. Two weeks after the EBRT was completed, an interstitial high-dose-rate brachytherapy of the cervix uteri was initiated, delivering two doses of 9 Gy in a period of two weeks, while uterus body/fundus laid outside of the 7.2 Gy isodose across multiple planes, showing a steep intrauterine gradient with higher dose at the cervix and the lower uterine segment, and lower dose toward the fundus.

**Figure 4:**
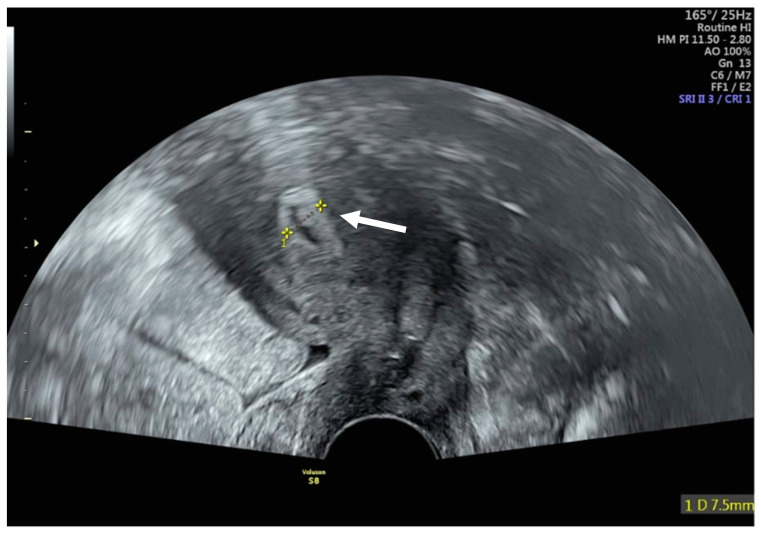
Transvaginal ultrasound demonstrating preserved endometrial function. Transvaginal ultrasound performed in the follicular phase shows an endometrial thickness of 7.5 mm (white arrow), consistent with preserved endometrial function.

Remarkably, menstruation resumed seven months after treatment completion, with regular cycles of 27–30 days and a bleeding duration of approximately three days, without intermenstrual bleeding. The early follicular-phase serum hormone measurement on cycle day 3 revealed an affected ovarian reserve, as indicated by an anti-Müllerian hormone (AMH) level of 0.3 ng/mL, and partial ovarian function with follicle-stimulating hormone (FSH) levels of 13.5 U/L (follicular reference 3–10 U/L), luteinizing hormone (LH) levels of 12 U/L (follicular reference 2–12 U/L) and estradiol (E2) at 221 pmol/L (follicular reference 70–500 pmol/L). An antral follicle count (AFC) and longitudinal serial hormone measurements were not systematically performed. The transvaginal ultrasound in the proliferative phase showed an endometrial thickness of 7.5 mm, consistent with preserved endometrial function (Figure 4). These findings, combined with the regularity of menstruation, indicate partial endometrial and ovarian preservation despite reduced ovarian reserve.

**Figure 5:**
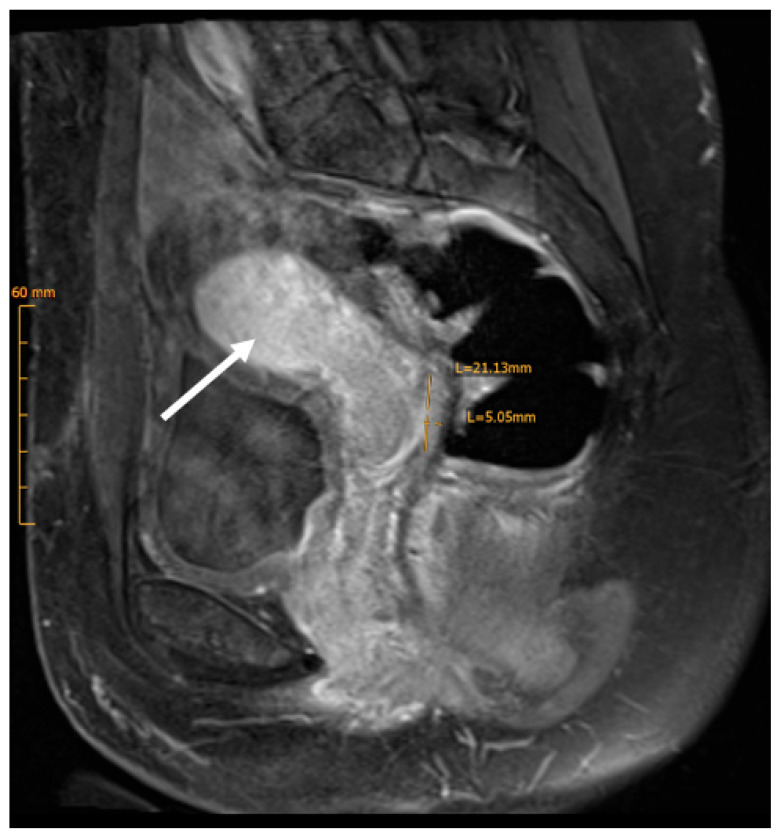
Postoperative MRI demonstrating post-radiation uterine changes. MRI shows a preserved uterine contour with diffusely low T2 signal in the myometrium and partial blurring of the junctional zone, compatible with post-radiation change, as well as a thin endometrium (white arrow).

**Figure 6:**
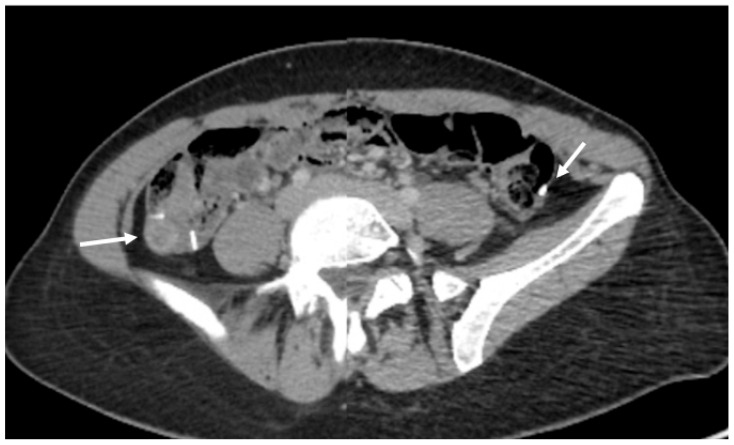
Postoperative pelvic MRI showing radiopaque ovarian clips. Pelvic MRI demonstrates radiopaque clips (white arrows) placed on each ovary (two on the right side and one on the left) for postoperative localization.

The follow-up Pap smears were normal, although HPV high-risk types 59 and 58 persisted. The postoperative MRI demonstrated a preserved uterine contour with diffusely low-T2 myometrium and partial junctional-zone blurring, compatible with post-radiation change, and a thin endometrium (Figure 5). The PET–CT follow-up was normal, without any signs of recurrent tumor (Figure 6). Over a two-year follow-up with six-monthly clinical evaluations, the patient has remained disease-free and is now undergoing oncofertility counseling with reassessment of the ovarian reserve, confirmation of the feasibility of oocyte retrieval from the transposed ovaries, controlled ovarian stimulation, and oocyte cryopreservation.

## 3. Discussion

Fertility preservation in cancer patients with advanced-stage disease presents a significant challenge in gynecological oncology. A primary CCRT with external beam radiation and cisplatin-based agents, accompanied by a brachytherapy regimen, is the treatment of choice in such cases, conferring a high risk of premature ovarian insufficiency and affecting endometrial receptivity [7]. Techniques like OT are essential in pre-treatment planning for younger patients with FIGO stage IIIC1 cervical cancer in the setting of preservation of ovarian endocrine function and reproductive potential [12].

The impact of chemotherapeutic drugs on ovarian function varies, depending on the chemotherapeutic agent, the dosage and age of the patient [8]. Chemotherapeutic drugs inhibit angiogenesis and DNA methylation, causing ovarian tissue ischemia, follicular apoptosis and destruction of larger growing follicles [13]. This damage results in the depletion of AMH and activation of premodial follicles, which are then not replaced, leading to ovarian failure [14]. More specifically, platinum agents like cisplatin may damage oocytes, activating proapoptotic promoters and damaging DNA [8,15]. Radiation also has a significant impact on oocyte function, as oocytes are sensitive to it. High doses of uterine radiation lead to irreversible vascular and morphological oocyte damage [13]. The estimated dose of radiotherapy leading to lethal ovarian damage and to primary ovarian failure is 14.3 Gy in 30-year-old patients [8]. The radiation dose required to induce ovarian failure decreases with increasing age, underlining age as a crucial factor in treatment considerations. Radiation below 4 Gy does not appear to affect fertility [14].

The clinical justification for OT in FIGO IIIC1 disease is primarily endocrine preservation rather than the assurance of future uterine fertility. Pelvic CCRT typically impairs endometrial receptivity and uterine vascular compliance, making the likelihood of uterine fertility low. However, OT can still be clinically meaningful by reducing ovarian dose and preserving hormonal function, thereby preventing or attenuating premature ovarian insufficiency and its associated morbidity. Systematic reviews in cervical cancer patients undergoing OT prior to pelvic radiotherapy consistently report meaningful rates of ovarian function preservation [9,10]. Age over 25 years and radiation total dose to the ovaries > 5 Gy are reported as risk factors for ovarian failure [16]. While various therapeutic age cut-off limits are reported, the current consensus indicates that women up to 35 years benefit significantly from OT [17]. Lateral OT is associated with more favorable results, as the ovaries are positioned as far away as possible from the radiation field, allowing the maintenance of their hormonal function and the preservation of endogenous estrogen production [18]. When transposed ovaries are out of transvaginal reach, transabdominal ultrasound-guided oocyte retrieval enables ART access [19]. Cryopreservation remains a standard option according to the ESGO/ESTRO/ESP and the ESGO/ESHRE/ESGE guidelines [6]. These guidelines also emphasize early oncofertility referral, individualized consideration of ovarian transposition when radiotherapy is planned, and structured post-treatment surveillance.

Based on this knowledge, lateral OT was also performed in our case, achieving the maintenance of the ovarian hormonal function of the patient, as indicated by FSH, LH and estrogen levels. Several factors could explain the preserved ovarian function in our patient. The dose heterogeneity and partial ovarian shielding are credible contributors. After transposition of the ovaries to the paracolic gutters, the effective ovarian dose is reduced. In our patient, a DVH analysis showed right ovary D_mean: 1.2 Gy/D_max 1.9 Gy and left ovary D_mean 1.1 Gy/D_max 1.7 Gy, which are below the commonly cited thresholds for endocrine preservation [20]. Moreover, the distance between the lower pole of the ovaries and the pelvic PTV, which was more than 4.0 cm, strengthens the association between the surgical transposition technique and the very low ovarian doses observed. Cisplatin-based chemotherapy may also play a major role in terms of ovarian shielding as it tends to damage the growing follicles while sparing parts of the primordial pool, contributing to endocrine rebound [21].

Importantly, AMH primarily reflects quantitative ovarian reserve (follicle pool) and does not directly measure ongoing cyclic endocrine activity; therefore, regular cycles can occur despite markedly reduced AMH. In this case, endocrine assessment was performed at a single documented post-treatment timepoint (cycle day 3) together with a follicular-phase ultrasound; antral follicle count (AFC) and longitudinal serial hormone measurements were not systematically collected during routine oncology follow-up and are addressed as limitations.

OT in cervical cancer requires careful selection. Ovarian metastasis is reported to be uncommon in squamous cell carcinoma compared with adenocarcinoma, and systematic reviews suggest OT can be oncologically acceptable when ovaries appear normal and there is no evidence of adnexal disease [9,10].

Despite preoperative MRI/PET-CT suggesting pelvic nodal involvement, the key management question prior to definitive CCRT was, in our case, whether para-aortic lymph nodes (PALN) were involved, because PALN metastasis would justify extended-field radiotherapy and may have altered overall prognosis and treatment planning. While PET/CT is pivotal for nodal assessment in patients with locally advanced cervical cancer, multiple analyses show a moderate sensitivity to false negative rates for para-aortic disease as high as 20%, particularly for microscopic metastases [11,22]. In this context, the updated ESGO/ESTRO/ESP 2023 guidelines allow selective para-aortic surgical staging in pelvic-node-positive patients with negative para-aortic imaging [6]. In line with the current evidence, we therefore performed a laparoscopic para-aortic lymph node resection to individualize field design and avoid geographic miss or unnecessary extended-field irradiation.

Radiation also affects the uterus’s function, reduces vascularity, leads to endometrial fibrosis and insufficiency, while direct radiation leads to endometrial atrophy. There are limited data on the critical dose of radiation affecting endometrial function. According to the existing literature, a dose of more than 30 Gy may lead to endometrial dysfunction, while a dose of more than 45 Gy is incompatible with a future pregnancy [23,24]. Although radiation doses of more than 45 Gy are linked to endometrial dysfunction, there are studies reporting residual function in the endometrium, appearing as hematocolpos and hematometra or irregular vaginal bleeding, after a curative radiotherapy of up to 80 Gy [25]. McKay et al. also described cyclical vaginal bleeding in 23% of premenopausal cervical cancer patients post RT after the initiation of hormone replacement therapy (HRT) [26]. This temporary uterine activity may represent the gradual decline and apoptosis of endometrial cells post-treatment [26], or this residual endometrial function may be attributed to the uneven radiation dose distribution in brachytherapy, responsible for sparing certain areas of the uterus, like the uterine fundus and cornual regions [26,27,28]. Histological assessment has also shown that proliferation of the endometrium can still be observed after RT, further supporting these findings. Likewise, the rapid dose fall-off in brachytherapy likely spares the uterine fundus and segments of the endometrium, permitting cyclical bleeding despite high cervical doses [29]. Moreover, patient-specific radiosensitivity varies with age, and younger women may better tolerate limited exposure and may show a vascular/stromal recovery sufficient to restore endocrine activity [30].

In our case, the entire uterus was encompassed within the PTV. Consequently, all uterine substructures received the full prescribed dose (54 Gy) without any sparing. In interstitial HDR brachytherapy (9 Gy × 2), the cervix was encompassed by the 9 Gy isodose, whereas the uterine body/fundus lay largely outside the 7.2 Gy isodose in multiple planes, indicating substantially lower fundal doses. The endometrium showed a heterogeneous pattern with higher doses close to the applicator and lower doses toward the fundus. This steep dose fall-off provides a plausible explanation for the retained cyclic endometrial pattern.

In our patient, high-risk HPV (types 58/59) remained detectable at follow-up despite normal cytology. Persistent HPV DNA after CCRT has been reported as a potential risk marker associated with higher rates of recurrence, whereas early post-treatment HPV clearance correlates with better local control and survival [31]. However, HPV positivity should be carefully interpreted in the context of clinical findings. A growing body of literature suggests that post-treatment HPV vaccination may also play a crucial role, as it may reduce the recurrence of HPV-related disease, especially when related to HPV 16 or HPV18, in women treated with local excision [32,33]. However, the quality of the evidence is low, especially in women with locally advanced cervical cancer treated with CCRT, and therefore, a higher level of evidence is still needed from randomized trials. Accordingly, we adopted closer surveillance, in line with ESGO/ESTRO/ESP guidance, incorporating HPV-based testing at 6–12 and 24 months. Moreover, after counseling regarding the potential benefits and limitations, and in the absence of prior HPV vaccination, the patient elected to receive prophylactic HPV vaccination as part of her post-treatment care [6].

An interesting area of focus in our case, both from a diagnostic standpoint and as a potential adjunct for post-treatment surveillance of tumor control, is the role of serum tumor markers in cervical cancer. In cervical squamous cell carcinoma (SCC), squamous cell carcinoma antigen (SCC-Ag) is the most commonly reported marker and has been associated with parametrial invasion, nodal metastases, and recurrent risk, whereas CA-125, a marker more commonly used in ovarian cancer, may be measured in some settings but remains nonspecific for cervical SCC [34,35]. In our case, serum tumor markers were not routinely obtained; therefore, post-treatment surveillance relied on clinical evaluation, imaging, and HPV testing. Nevertheless, baseline SCC-Ag levels could be useful as an adjunct alongside MRI to better contextualize disease extent and overall tumor burden and to support longitudinal assessment during follow-up [34,35].

Beyond tumor markers, angiogenesis also plays a key role in cervical cancer biology. The vascular endothelial growth factor (VEGF) is a mediator of tumor angiogenesis supporting tumor growth and metastatic potential. VEGF expression is upregulated in cervical cancer and has been associated with an unfavorable prognosis, with higher VEGF levels correlating with poorer survival outcomes [36,37]. Although VEGF was not assessed in our case, it may be considered an adjunct prognostic biomarker in selected patients.

The therapeutic relevance of VEGF in cervical cancer is underscored by bevacizumab, a monoclonal antibody targeting VEGF that inhibits angiogenesis. In patients with metastatic or recurrent/persistent cervical cancer, the phase III GOG240 trial showed improved clinical outcomes when bevacizumab was added to platinum–paclitaxel chemotherapy [38]. Bevacizumab is an important targeted agent in cervical cancer, with demonstrated benefit in metastatic or recurrent/persistent disease when used in combination with chemotherapy, although this benefit was accompanied by higher rates of adverse events such as hypertension, fistula formation, and thromboembolic complications [38].

The ongoing ovarian function and menstruation in our case are encouraging, but do not guarantee fertility. Our patient’s low AMH (0.3 ng/mL) suggests a reduced ovarian reserve, while the oocyte competence was not tested, and response to stimulation remains unknown. Also, the fact that no antral follicle count (AFC) and longitudinal serial hormone measurements were performed is an important limitation. From the uterine perspective, radiation can reduce uterine perfusion and promote endometrial fibrosis, impairing implantation and placentation despite cyclical endometrial changes. Even though the sonographic findings with an endometrium of 7.5 mm in the follicle phase indicate a preserved endometrial function, its receptivity is not guaranteed. A further limitation is that ovarian and endometrial function were assessed using surrogate markers (menstrual cyclicity, a single early follicular endocrine panel, and endometrial thickness). Even though this case shows hormonal and endometrial activity, the true probability of conception and live birth after full-dose pelvic chemoradiotherapy remains uncertain and warrants individualized evaluation and counseling. As the patient is now undergoing specialized oncofertility, counseling with planned assisted reproduction, repetition of the endocrine testing (AMH/FSH/LH/estradiol) and AFC assessment are planned as part of the standard fertility work-up. Moreover, uterine evaluation will be individualized (e.g., cycle-timed ultrasound ± Doppler and, if indicated, cavity assessment).

## 4. Conclusions

This case illustrates that menstrual and partial endocrine function may persist after CCRT for FIGO stage IIIC1 cervical cancer when ovarian transposition and careful radiotherapy planning minimize ovarian and uterine dose exposure. To our knowledge, preservation of regular menstruation following lateral ovarian transposition and primary chemoradiotherapy in FIGO IIIC1 disease is very sparsely reported, as such regimens are typically associated with ovarian failure and endometrial damage. In this context, the present case is notable for the coexistence of three findings that are rarely reported together: (i) extremely low ovarian radiation exposure achieved through lateral ovarian transposition, (ii) biochemical and clinical evidence of preserved ovarian endocrine function, and (iii) sustained cyclic endometrial activity manifested by regular menstruation despite full-dose pelvic external beam radiotherapy and brachytherapy.

While preserved menstruation is encouraging, it does not necessarily imply preserved fertility, and reproductive potential after full-dose pelvic chemoradiotherapy should be individualized. Prospective studies are therefore required to evaluate reproductive endpoints alongside oncologic safety. Standardized assessments should include hormonal assessment, pelvic imaging, and DVH-based uterine/ovarian dosimetry to define the conditions under which endocrine and reproductive function may be preserved after chemoradiotherapy.

## Data Availability

The clinical data presented in this article are not readily available to maintain the privacy of the treating physicians and their patient.

## Abbreviations

The following abbreviations are used in this manuscript:

AFC	antral follicle count
AMH	Anti-Müllerian hormone
CCRT	Concurrent Chemoradiotherapy
DVH	Dose-volume histogram
E2	Estradiol
EBRT	External beam pelvic radiotherapy
FIGO	International Federation of Gynecology and Obstetrics
FSH	Follicle-stimulating hormone
HDR	high-dose-rate
HPV	Human Papillomavirus
HRT	hormone replacement therapy
IMRT	intensity-modulated radiotherapy
LH	Luteinizing hormone
MRI	Magnetic Resonance Imaging
OT	Ovarian transposition
PALN	para-aortic lymph nodes
PET-CT	Positron Emission Tomography–Computed Tomography
SCC-Ag	squamous cell carcinoma antigen
SCC	squamous cell carcinoma
VEGF	vascular endothelial growth factor
